# Molecular mechanisms underlying the effectiveness of triiodothyroacetic acid (Triac) in patients with resistance to thyroid hormone β (RTHβ): a multicentre, international, retrospective cohort study

**DOI:** 10.1016/j.ebiom.2026.106512

**Published:** 2026-10-14

**Authors:** Wenjun Liao, Federica Marelli, Carla Moran, Irene Campi, Stefan Groeneweg, Zainaba Mohamed, Eman Kamaleldeen, Raja Padidela, Catharina Olivius, Luuk Oosterkamp, Selmar Leeuwenburgh, Greta Lyons, Robin P. Peeters, Krishna Chatterjee, Luca Persani, Marcel Meima, W. Edward Visser

**Affiliations:** a Department of Internal Medicine, Thyroid Center, Erasmus Medical Center, Rotterdam, the Netherlands; b Department of Endocrine and Metabolic Diseases, IRCCS Istituto Auxologico Italiano, Milan 20149, Italy; c Endocrine Section, Beacon Hospital, Dublin D18 AK68, Ireland; d Endocrine Department, St. Vincent’s University Hospital, Dublin D04 T6F4, Ireland; e School of Medicine, University College Dublin, Dublin D04 C1P1, Ireland; f College of Medicine and Health, University of Birmingham, Birmingham, UK; g Birmingham Women’s and Children’s NHS Foundation Trust, School of Medical Sciences, Birmingham, UK; h Department of Endocrinology, Birmingham Women’s and Children’s NHS Foundation Trust, Birmingham, UK; i Royal Manchester Children’s Hospital and Manchester Academic Health Science Centre, Faculty of Biology, Medicine and Health, The University of Manchester, Manchester, UK; j Department of Medicine, Hospital of Halland, Tölövägen 3, Kungsbacka 434 80, Sweden; k Institute of Metabolic Science, University of Cambridge, Cambridge CB2 0QQ, UK; l Department of Medical Biotechnologies and Translational Medicine, University of Milan, Milano 20100, Italy

**Keywords:** Resistance to thyroid hormone beta, RTHβ, Thyroid hormone receptor, Triac, Tiratricol

## Abstract

**Background:**

Disease features in resistance to thyroid hormone beta (RTHβ), caused by *THRB* mutations, arise from insensitivity to thyroid hormone in tissues expressing thyroid hormone receptor (TR)β and from lifelong thyrotoxic effects in TRα1-expressing tissues, posing substantial cardiovascular morbidity/mortality. The T3-analogue triiodothyroacetic acid (Triac) holds potential as an effective treatment. Systematic data on Triac therapy in RTHβ are lacking, and the mechanisms through which Triac acts are unclear. This study aims to elucidate these knowledge gaps.

**Methods:**

Clinical and biochemical data were obtained through an, international retrospective cohort study in patients with RTHβ. Transcriptional activity of all TRβ mutants, on the background of the pituitary-expressed β2 receptor isoform and positively (DR+4-TRE) or negatively (TSHα-TRE) regulated reporter genes plus T3 or Triac, was studied. Thyroid function and TSH responses to T3 and Triac were tested in zebrafish expressing TRβ mutants.

**Findings:**

We collected data from 28 patients with RTHβ (range: 0.4–73.1 years) with 18 unique mutations treated with Triac in 6 centres. Triac reduced TSH (by 43%) and FT4 (by 37%) concentrations in all heterozygous patients, supported by consistent findings in zebrafish. Our functional studies showed that TRβ2 mutants exhibited reduced (all) or absent (G432del) T3-dependent transcriptional activity, with similar EC50 and IC50 values for T3 and Triac, except for R316H, which showed a 5-times lower EC50/IC50 value for Triac compared to T3.

**Interpretation:**

In this international, multicentre study we have documented that Triac lowers elevated FT4 concentrations in patients with RTHβ effectively. Integrating our clinical and molecular observations we suggest that Triac can act via different modes of action, with activation of the wild-type receptor being a relevant mechanism. Our study may inform clinical management and could shape future interventional studies in patients with RTHβ.

**Funding:**

Financial support is acknowledged by WL (10.13039/501100015375Affiliated Hospital of Southwest Medical University) WEV (10.13039/501100001826ZonMw (Dutch Research Council, VIDI 09150172210071)), KC (10.13039/100004440Wellcome (210755/Z/18/Z), 10.13039/501100018956NIHR Cambridge Biomedical Research Centre (NIHR20312) and 10.13039/501100000265Medical Research Council United Kingdom (MRC_MU_UU_00014/40)), LP (European Union - Next Generation EU - NRRP M6C2 - Investment 2.1 Enhancement and strengthening of biomedical research in the NHS (code: PNRR-MR1-2022-12375726).

**Research in context**

**Evidence before this study**

Resistance to thyroid hormone beta (RTHβ) is a rare disorder caused by TRβ mutations, resulting in elevated thyroid hormone with non-suppressed TSH concentrations. The thyroid hormone analogue Triac has potential to ameliorate the thyrotoxicosis in RTHβ. We searched PubMed for studies published in English, using the search terms “Resistance to thyroid hormone beta”, “RTHβ”, “Triac”, “tiratricol”, “triiodothyroacetic acid”, and “mechanisms”. Previous clinical studies were limited to case reports and small series, and molecular studies were insufficient to understand the potential mechanisms of action for Triac in RTHβ.

**Added value of this study**

To our knowledge, this international, multicentre cohort study, including 28 patients with 18 different TRβ mutations and from 6 sites, represents the largest clinical study in which the effects of Triac in patients with RTHβ were evaluated. Our data indicate that Triac decreases elevated thyroid hormone concentrations effectively. Integration of our clinical observations and molecular findings cell and zebrafish models suggest that Triac acts via different modes of action, with activation of wild-type TRβ being a relevant mechanism.

**Implications of all the available evidence**

Our results support Triac as an effective therapy for RTHβ across a wide range of mutations, particularly in heterozygous patients where wild-type receptor activation contributes to Triac’s efficacy. These insights refine the mechanistic understanding of Triac’s action and justify prospective interventional trials to evaluate broader clinical outcomes, including cardiovascular and metabolic effects.

## Introduction

Thyroid hormones regulate different physiological processes including growth, metabolic rate, cardiac function and development of the central nervous system.^1^ The genomic effects of the active hormone triiodothyronine (T3) are mediated through binding to thyroid hormone receptors (TRs). TRs are ligand-dependent transcription factors, which bind to thyroid hormone response elements (TREs) in the promoter regions of target genes. Unliganded TRs are bound to corepressors (e.g., nuclear receptor corepressor (NCoR) or silencing mediator for retinoic acid and thyroid receptors (SMRT)), thereby repressing gene transcription. T3 occupancy of TR results in dissociation of corepressors and recruitment of coactivators (e.g., steroid receptor coactivator-1 (SRC-1)), enabling transcriptional activation.^2^

There are three highly homologous T3-binding TR isoforms: TRα1, encoded by the *THRA* gene, and TRβ1 and TRβ2, isoforms encoded by the *THRB* gene.^2^ TRα1 is mainly expressed in cardiac and skeletal muscle, bone, gastrointestinal tract and central nervous system; TRβ1 is mainly expressed in liver and kidney, while TRβ2 is expressed in hypothalamus, pituitary, cochlea and retina.

Resistance to thyroid hormone beta (RTHβ), typically caused by heterozygous *THRB* mutations, occurs in ∼ 1 in 20,000–40,000 individuals.^3^^,^^4^ Mutations localize to distinct hotspot areas in the ligand binding domain of TRβ. TRβ mutants inhibit wild-type receptors in a dominant negative manner, likely through failure of corepressor dissociation from mutant TRβ thereby repressing T3-target gene expression. Patients with RTHβ exhibit elevated serum (F)T4 accompanied by non-suppressed TSH concentrations, reflecting resistance to thyroid hormones at the pituitary level. Increased liver fat and dyslipidaemia result from hepatic resistance to thyroid hormones.^5^ TRα-expressing tissues (e.g., the heart) retain normal sensitivity and are exposed to increased circulating thyroid hormone concentrations. Recently, the consequences of cardiac manifestations in RTHβ have been reported in two independent cohorts.^6^^,^^7^ Atrial fibrillation occurred ∼10-times more frequently in RTHβ compared to the general population and cardiovascular morbidity and mortality were ∼3-times increased.^6^^,^^7^ Thus, the lifelong thyrotoxic state in TRα-expressing tissues together with a hypothyroid state in TRβ-expressing tissues result in diverse phenotypes and increased morbidity and mortality.

Unfortunately, therapeutic options in RTHβ are limited as antithyroid drugs, thyroid surgery or radioiodine treatment are unable to effectively reside euthyroidism in all tissues.^7^ Palpitations can be managed symptomatically by beta blockade, but it is unclear whether they improve cardiovascular morbidity and mortality.^8^ Moreover, beta-blockers do not target non-cardiac disease features.

Recently, the T3 analogue 3,5,3’-triiodothyroacetic acid (Triac or tiratricol), which has an increased affinity for TRβ above T3, was approved by the European Medicine Agency for MCT8 deficiency, a rare metabolic and developmental disorder due to a defect in the thyroid hormone transporter MCT8 as it was able to effectively and safely ameliorate peripheral thyrotoxicosis seen in this condition.^9^^,^^10^ In case reports and small case series Triac has been reported to reduce thyroid hormone concentrations in patients with RTHβ.^11^ However, systematic evaluation of Triac therapy on thyroid function tests in patients with RTHβ is lacking and the molecular mechanisms underlying its potential effectiveness are unclear.

Here, we document the effectiveness of Triac on thyroid function tests and clinical and other biochemical outcomes in a multicentre, international cohort of patients with RTHβ. We investigated the mechanism of action mediating actions of Triac with all TRβ mutants present in these cases. Our findings inform clinical management of RTHβ and may guide future clinical trials in this rare disorder.

## Methods

### Study design and participants

In this multicentre, international RTHβ retrospective cohort study, data were collected from patients with a genetically proven pathogenic variant in *THRB* who received Triac at 6 sites (Birmingham, 1 patient; Cambridge, 11 patients; Kungsbacka, 5 patients; Manchester, 1 patient; Milan, 6 patients; Rotterdam, 4 patients) in 4 countries in Europe. No other inclusion or exclusion criteria were used. No *a priori* power calculation was performed given the pragmatic, retrospective design of the study.

### Ethical considerations

This study conforms to the Declaration of Helsinki, Good Clinical Practice guidelines and was evaluated and approved by the appropriate local institutional review boards or ethics committees. However, for the retrospective analysis of existing datasets of patients in routine clinical care, the majority of centres did not require additional specific institutional review board approval. For other centres, studies were either ethically approved (Ethic Committee of the IRCCS Istituto Auxologico Italiano (code: PNRR-MR1-2022-12375726)) or the ethics committee provided a waiver for approval (Erasmus MC, MEC-2015-362). Informed consent was obtained from the parents or legal representatives of all enrolled patients, unless the relevant institutional review board and/or local regulations had authorized the use of anonymised patient data without additional consent.

The zebrafish studies were approved by the Animal Welfare Body (OPBA) of the University of Milan, Italy (protocol 198283). All experiments followed EU regulations on laboratory animals (Directive 2010/63/EU).

### Procedures

For all patients enrolled, age, sex assigned at birth and *THRB* mutations were documented. Measurements prior to commencement of Triac and last available data following therapy were retrieved from their medical records: body weight, heart rate, blood pressure, use of antithyroid drugs or proton pump inhibitors, Triac dose and duration of treatment, and thyroid function tests (TSH and FT4 concentrations) and, when available, other biochemical tests (sex hormone binding globulin (SBHG), ferritin, total cholesterol, low-density lipoprotein (LDL), bone alkaline phosphatase, procollagen type I N-propeptide (P1NP) beta-CrossLaps (CTx), TPO-antibodies) were measured in each centre according to local procedures. Some baseline measurements from 6 patients have been reported before.^12^ The Jostel’s TSH index (TSHI), Thyrotroph Thyroid Hormone Sensitivity Index (TTSI) and SPINA-GT (thyroid’s secretory capacity) were calculated.^13^

### Plasmid construction of wild-type or mutants TRβ and other vectors

The pcDNA3-FLAGTRβ1 plasmid has been described previously.^14^ The human TRβ2 cDNA was amplified and subcloned into the EcoRI and Xbal sites of the vector pcDNA3 fused at the 5’-FLAG-tag encoding sequence and an optimized Kozak sequence (see Supplementary Table S1 for primers). The patients’ mutations were introduced using the QuikChange II Mutagenesis kit (200521, Agilent Technologies, Amstelveen, The Netherlands) according to manufacturers’ protocol (see Supplementary Table S2 for primers). Sequences of mutant constructs were confirmed by Sanger sequencing. The luciferase reporter plasmids pA3LUC--MALtk-luciferase containing a positive TRE with a DR+4 configuration (MAL-TRE) and - pA3LUC-TSHαtk-luciferase containing a negative (TSHα-TRE) have been described previously; these vectors contain three SV40 polyA cassettes to eliminate read through transcription from the vector.^15^

### Cell culture and transfection

JEG3 cells (RRID:CVCL_0363) authenticated by STR profiling by ECACC; mycoplasm testing was negative. JEG3 cells were maintained in DMEM/F12 medium with GlutMAX™ (31331093), containing 9% foetal bovine serum (FBS) (A5256702),100 U/mL penicillin, and 100 μg streptomycin (15070063, all from ThermoFisher Scientific, Waltham, MA, USA). Cells were plated in 24-well plates for luciferase reporter assays (TSHα-TRE) or 96-well plates (MAL-TRE) for luciferase reporter assays, and in 6-well plates for Western blot analysis and transfected using Xtreme Gene 9 transfection reagent (06365809001, Roche Diagnostics, Almere, NL) according to the manufacturer’s protocol. Cells cultured in 24-well plates were transfected with a mixture of: 20 ng of pCDNA3-FLAGTRβ2 (wild-type or mutant) or the pCDNA3 empty vector control; 60 ng of pMaxGFP; and 120 ng of the negative reporter plasmid pA3LUC-TSHαtk-luciferase. Transfection was performed in DMEM/F12 medium supplemented with 9% charcoal-stripped FBS. Similarly, cells in 96 wells plates were transfected in DMEM/F12 medium supplemented with 9% charcoal-stripped FBS with 10 ng pCDNA3-FLAGTRβ2 (wild-type or mutant) or pCDNA3 as empty vector control, 30 ng pMaxGFP and 60 ng positive reporter plasmid pA3LUC-MALtk-luciferase for transcriptional activity. After 24 h of transfection, the cells were stimulated with DMEM/F12 medium supplemented with 0.1% bovine serum albumin and increasing concentrations of Triac (T7650) or T3 (T2877, both from Sigma–Aldrich, St. Louis, MO, USA) (0–10,000 nM) for 24 h, after which cells were lysed and measured for luciferase activity.

### Luciferase assay

Luciferase activity was measured as previously described.^16^ To correct for variations in transfection efficiency, the ratio of luciferase to GFP activity was calculated. The log half maximal effective concentration (logEC50) for MAL-TRE, log half maximal inhibitory concentration (logIC50) for TSHα and maximal response values were obtained from non-linear regression analysis from individual experiments using GraphPad Prism version 9.0.0. Luciferase values were normalized as percentage of the maximal response values observed in cells expressing wild-type TRβ2 stimulated with T3. Results are expressed as mean ± standard error of the mean (SEM) of at least three independent experiments performed in triplicate.

### Zebrafish husbandry and maintenance

Wild-type AB adult zebrafish (ZIRC_ZL1) were maintained and bred for multiple generations in the institutional zebrafish facility of the University of Milan under standard housing conditions. Embryos were obtained by natural spawning and cultured until the desired developmental stages based on established morphological criteria.

### Experimental strategy for zebrafish studies

The overall experimental strategy for the zebrafish studies was done as has been described.^17^^,^^18^ All zebrafish experiments were performed using biological replicates derived from independent injections and multiple clutches. Key design features, including pooling strategies, developmental stages, and approximate subgroup sample sizes, are summarized in Supplementary Table S4.

Sample configuration was defined based on assay-specific requirements, including RNA yield, detection sensitivity, and inter-replicate variability. Embryos with severe developmental delay or malformations were excluded prior to pooling. Injection efficiency was verified by co-injection of a fluorescent tracer, and only embryos displaying homogeneous tracer distribution were included in the analyses. Embryos were randomly allocated to experimental conditions. Formal blinding was not applied; however, outcome measures were obtained using objective instrument-based quantification and predefined analysis pipelines. Because pooled larvae were used as the experimental unit, formal power calculations based on individual-level variability were not applicable.

### RNA microinjection and chemicals treatment

Wild-type and mutated (MUT) RNAs were *in vitro* transcribed using mMessage mMachine T7 Ultra kit (AM1345, ThermoFisher Scientific, Waltham, MA, USA), following the manufacturer’s instructions. To minimize off-target effects, TRβ1 and TRβ2 mRNAs (wild-type, R316H, G344E and G432del) were injected at a dose of 50 pmol/embryo. To monitor injection efficiency, mRNA was co-injected with Rhodamine B isothiocyanate–Dextran (D3308, Thermo Fisher Scientific, Waltham, MA, USA). Embryos were screened at the one-cell stage for homogeneous tracer distribution, and only normally developing embryos with adequate tracer signal were included in subsequent analyses. Following a previously published protocol^19^ starting from 20 to 24 h post fertilization (hpf), embryos were harvested in fish water (FW, 0.01 mg Istant Ocean, 0.019 mg MgCl2, and 0.01 Nacl in ddH2O) containing 0.002% of 1-phenyl-2-thiourea (P7629, ≥98% purity, Sigma–Aldrich, St. Louis, MO, USA) to prevent pigmentation and 0.01% methylene blue (C.I. 52015, Sigma–Aldrich, St. Louis, MO, USA)to prevent fungal growth. T3 (6666/50, ≥98% purity, BioTechne, Abingdon, UK), and Triac (S4185, ≥98% purity, Selleck Chemicals, Houston, TX, USA) were resuspended in DMSO (50 mg/mL) and diluted in fish water at a desired concentration and administered to the embryos from the 6 up to 120 hpf.

### RNA extraction and qRT-PCR

Total RNA was extracted and retrotranscribed pooled embryos/larvae using standard TRIzol Reagent (15596026, Thermo Fisher Scientific, Waltham, MA, USA) and GoScript Reverse Transcription System (A5000, Promega Corporation, Madison, WI, USA). Relative mRNA expression was quantified by qRT-PCR and normalized against the housekeeping gene eef1a using the 2-ddCt method. Data represent mean ± standard deviation (SD). Primer sequences are listed in Supplementary Table S3.

### Fluorescent whole-mount *in situ* hybridization (FISH) and immunofluorescence (IF)

FISH experiments were performed using *tshba*-DIG riboprobe (FW: 3’-TCCAGACCCTCCAGACATCC-5’; REV: 3’-CAACCCTGCCAGTCAAACAC-5’), according to Thisse protocol.^20^ Larvae at 120 hpf were stained with Fast Blue B Salt 0.25 mg/mL Fast Blue BB (F3378, Sigma–Aldrich, St. Louis, MO, USA) and 0.25 mg/mL NAMP (N5000, Sigma–Aldrich, St. Louis, MO, USA) in SB8.2 (0.1 M Tris-HCl pH 8.2, containing 50 mM MgCl2, 100 mM NaCl, 0.1% Tween-20).

For IF, larvae at 120 hpf were fixed in 4% paraformaldehyde (158127, Sigma–Aldrich, St. Louis, MO, USA) overnight in PBT at 4 °C, and the immunostaining was performed according to the protocol described by He et al.^21^ To visualize T4-producing thyroid follicle, a rabbit anti-thyroxine BSA serum (1:500; 65–850, ICN Biomedicals, Costa Mesa, CA, USA), and Alexa Fluor 488-conjugated goat anti-rabbit IgG (AB_143165; 1:1000; A11008, Thermo Fisher Scientific, Waltham, MA, USA) were used.

For confocal acquisition of fluorescent signals, the heads were cut off and mounted in ventral orientation on glass slides and acquired using Nikon Eclipse Ti microscope (Tokyo, Japan) equipped with a 20× objective and laser-648 nm (for *tshba*), and laser-545 (for T4). To calculate *tshba*-cell volume, confocal images were analysed with Volocity Software (RRID:SCR_002668; PerkinElmer, Waltham, MA, USA) that quantify both number and intensity of fluorescent pixels on the same area and number of stacks for each sample, thus defining the cell-volume (μm3). FISH and IF experiments were performed using 15 embryos each, derived from three independent injections.

### Hormone measurement in zebrafish

Thyroid hormones (TT4, TT3, FT4, and FT3) and TSHβ concentrations were measured in homogenates of pooled larvae (N ∼ 200/replicate) using an enzyme-linked immunosorbent assay (ELISA).^19^ Briefly, pools of 200 larvae at 120 hpf were precisely scaled to obtain the wet weight (ww, g/ml), followed by homogenization in 400 μL of 1 × PBS by manual pipetting. After centrifugation (10 min at 13.000 rpm), and the supernatant was loaded into the microtiter plates. The levels of hormones were quantified using fish ELISA kits (Cusabio Biotech Co., Ltd., Wuhan, China) for TT4 (CSB-E08489f), TT3 (CSB-E08488f), FT4(CSB-EQF027512FI), FT3 (CSB-EQF027510FI), and TSHβ (CSB-EQ027261FI), and analysed with ViktorNivo Plate Reader (PerkinElmer, Waltham, MA, USA) set to 450 nm. The limits of detection were as follows: TT4, 20 ng/mL; T3, 0.5 ng/mL; FT4, 1.08 pmol/L; FT3, 0.38 pmol/L; TSHβ, 2.5 μIU/mL. The measurements of hormone levels were normalized to ww (g/ml), and to the number of larvae of each pool. The results were expressed as mean ± SEM of five pools/each, derived from at least 20 independent injections.

### *In silico* prediction of mutant TRβ function

YASARA Structure Software (YASARA Bioscience GmbH, Vienna, Austria)^22^ was used to model the TRβ1-G344E and R316H variants into a T3-liganded WT TRβ1 crystal structure (PDB-ID: 3GWS)^23^ and a Triac-liganded WT TRβ1 crystal structure (PDB-ID: 3JZC),^24^ using the side-chain substitution tool. Side-chain orientations were optimized using SCWALL (Side-Chain conformations With ALL available methods)^14^^,^^25^ after which the final models were minimized without further constraints. Finally molecular dynamic simulations were run for 1 ns in order to evaluate the predictive effects of both substitutions on ligand binding and overall protein structure. All images were created using YASARA Structure (Yasara Biosciences GmbH, Vienna, Austria) and Pov-Ray v3.6 software (www.povray.org, Indianapolis, IN, USA).

### Outcomes

We had two main outcomes. First, to compare the change in TSH and FT4 concentrations from baseline to the last available measurement in patients with genetically-confirmed RTHβ who were treated with Triac. We documented clinical (heart rate, blood pressure) and biochemical (SHBG, total cholesterol, LDL, CTx) outcomes. Second, we aimed to compare the *in vitro* responsiveness of Triac versus T3 in (i) cells expressing mutant versus wild-type TRβ and (ii) in zebrafish expressing mutant or wild-type TRβ.

### Statistical analysis

For outcomes in patients, p values and standard deviations (SDs) were calculated for the mean change from baseline to month 12 with the use of paired Student’s t-tests or Wilcoxon test when appropriate. Serum TSH concentrations were first log-transformed to normalize the distribution. For luciferase assays, the comparison between logIC50 or EC50 for Triac over T3 were determined by one-way ANOVA with Dunnett’s multiple comparisons test.

For FISH and IF experiments, and hormone measurements, the comparison between control and injected larvae was performed by One-way ANOVA (a) controls versus wild-type mRNAs; (b) controls vs wild-type vs mutant mRNAs; or Unpaired Student-t-test (c) controls vs THRB1 mutant vs THRB2 mutant. A p-value < 0.05 was considered statistically significant.

### Role of funders

The funders did not have any role in study design, data collection, data analyses, interpretation, or writing of report.

## Results

In 6 centres, 28 patients from 23 families were enrolled with a median age of 26.0 years (range: 0.4–73.1 years and 15 (54%) females with data captured between March 1st 2006 and July 26th 2024 (Table 1)). We documented 18 different heterozygous mutations and one patient carrying a homozygous R316H/R316H mutation. FT4 concentrations were elevated (1.8 times the ULN) with TSH concentrations being non-suppressed in all patients prior to Triac treatment. Auto-immune thyroid disease was present in one patient heterozygous for P452L, which explains the strongly elevated TSH, and four patients were concomitantly treated with carbimazole. The median duration of Triac treatment was 50 months (range: 5–118 months) with a median dose of 24 μg/kg/day (range: 11–61 μg/kg/day). Triac reduced TSH concentrations (3.23 ± 1.78 to 1.88 ± 0.76 mU/L, p < 0.0001) and FT4 concentrations (40.0 ± 13.5 vs 25.1 ± 10.6 pmol/L, p < 0.0001) in all heterozygous patients (Fig. 1A and B). The homozygous patient was excluded from this analysis, because both FT4 and TSH concentrations were above the upper limit of quantification at baseline; however, upon Triac treatment both TSH and FT4 concentrations were also clearly reduced. Triac treatment improved pituitary resistance as shown by the reduction in the Thyrotroph Thyroid Hormone Sensitivity Index (TTSI) (Fig. 1C) and pituitary output as shown by reduction in the Jostel’s TSH index (Fig. 1D), while thyroid secretory capacity (SPINA-GT) was marginally changed (Supplementary Fig. S1 and Supplementary Table S1).^26^ Triac reduced heart rate in patients (Table 2). The effects of Triac on blood pressure and biochemical markers reflecting thyroid status in liver and bone are documented in Table 2.

**Table 1:** Cohort characteristics.

	
Median age at start of Triac treatment (range; years; n = 28)	26.0 (0.4–73.1)
Sex (n = 28)	
Female	15 (54%)
Male	13 (46%)
Zygosity (n = 28)	
Heterozygous	27 (96%)
Homozygous	1 (4%)
Families (n = 23)	
With one affected member	19
With two affected members	3 (including 1 case of dizygotic twins)
With three affected members	1
Median duration of Triac treatment (months; n = 28)	50 (5–118)
Median Triac dose (range; μg/day; n = 27)	1400 [136–4200]
Median Triac dose (range; μg/kg/day; n = 27)	24 (8–61)
TPO positivity (n = 15)	5
Auto-immune hypothyroidism (n = 14)	0
Other medication (n = 28)	
Carbimazole	4

**Fig. 1:**
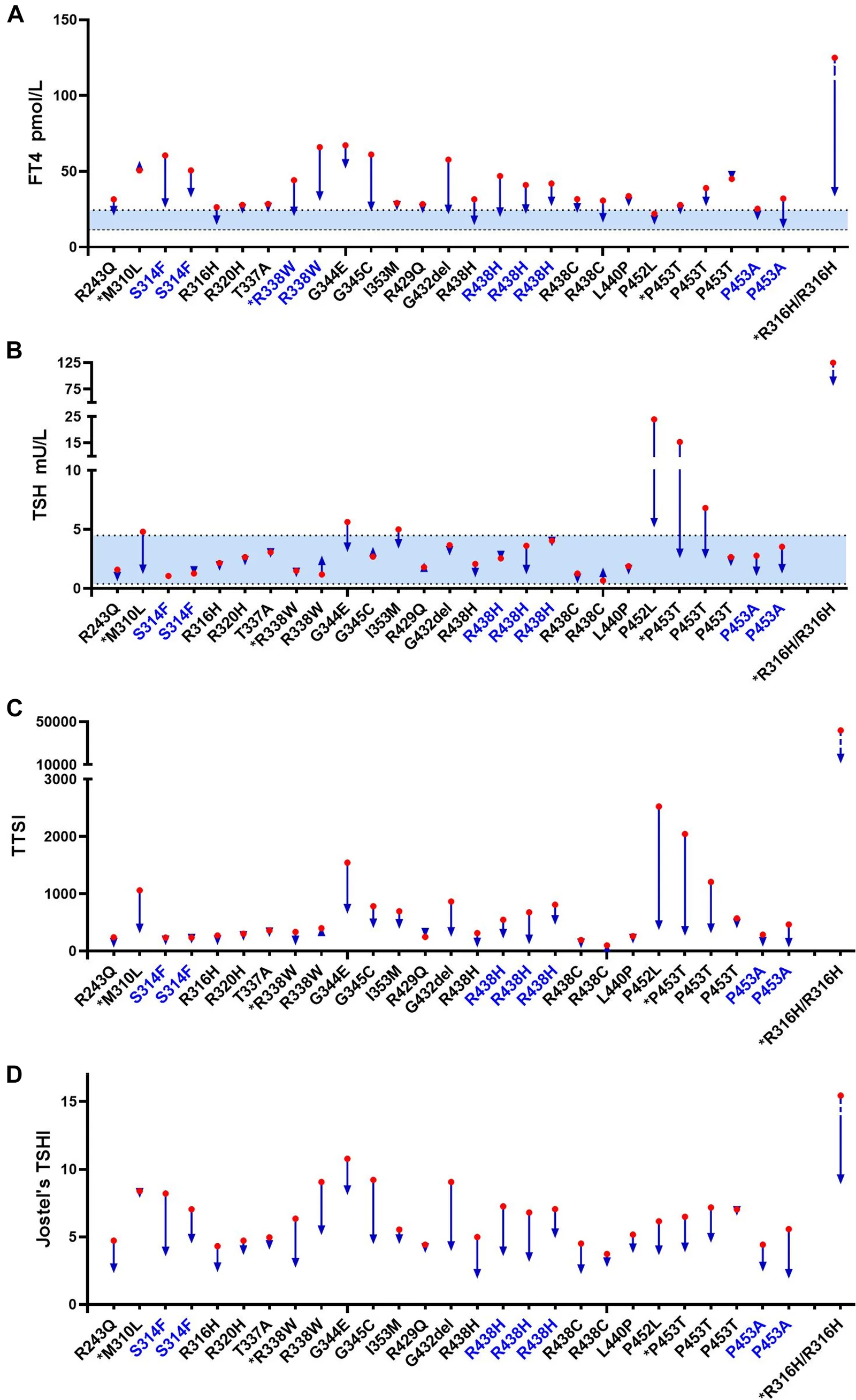
Thyroid function measurements, and pituitary indices in RTHβ patients prior to and during Triac therapy. Serum FT4 concentrations (A), TSH concentrations (B) TTSI (C) and Jostel’s TSHI (D) from baseline measurements prior to TRIAC treatment (red dot) to most recent measurements (blue arrow head). Patients belonging to a family with multiple participants denoted in blue. Asterisks indicate carbimazole use. The blue box indicates the reference interval.

**Table 2:** Effects of Triac on clinical and biochemical parameters.

	Baseline	Last measurement	p value
Primary outcomes			
TSH (mU/L) (n = 27)	3.23 ± 1.78	1.88 ± 0.76	**<0.0001**
FT4 (pmol/L) (n = 27)	40.0 ± 13.5	25.1 ± 10.6	**<0.0001**
Pituitary and thyroid indices			
Jostel’s TSHI (n = 27)	6.41 ± 1.82	3.94 ± 1.59	**<0.0001**
TTSI (n = 27)	652 ± 588	224 ± 158	**<0.0001**
SPINA-GT (n = 27)	6.91 ± 3.71	5.37 ± 2.94	**0.041**
Other clinical outcomes			
Heart rate (bpm) (n = 22)	86 ± 22	76 ± 13	**0.014**
Systolic blood pressure (mm Hg) (n = 23)	123 ± 17	122 ± 15	0.74
Diastolic blood pressure (mm Hg) (n = 23)	72 ± 13	71 ± 10	0.71
Other biochemical outcomes			
SHBG (relative to ULN) (n = 16)	0.47 ± 0.45	0.50 ± 0.40	0.27
Ferritin (relative to ULN) (n = 14)	0.36 ± 0.28	0.32 ± 0.18	0.64
Total cholesterol (mmol/L) (n = 19)	4.67 ± 1.11	4.45 ± 0.91	0.22
LDL (mmol/L) (n = 17)	2.92 ± 0.96	2.35 ± 0.78	**0.017**
Bone alkaline phosphatase (relative to ULN) (n = 12)	0.43 ± 0.11	0.48 ± 0.14	**0.031**
Beta cross laps (relative to ULN) (n = 10)	0.98 ± 0.24	1.00 ± 0.37	0.59
P1NP (relative to ULN) (n = 7)	0.79 ± 0.12	0.93 ± 0.25	0.38

A p value <0.05 was considered significant (values in bold).

Next, we performed *in vitro* studies to test the transcriptional activity of wild-type or mutant TRβ2 (the receptor isoform expressed in the pituitary) with increasing concentrations of T3 or Triac. For this, we cotransfected wild-type or mutant receptors into JEG-3 cells with a TRE-luciferase reporter construct for either a negatively (TSHα-TRE) or positively (MAL-TRE) T3-regulated gene. Fig. 2A and Supplementary Table S5 show variably impaired transcriptional activity of TRβ2 mutants using the TSHα-TRE reporter with both T3 and Triac. Marginally impaired IC50 values were seen for some mutants (e.g., for T3: wild-type 0.033 ± 0.004 *vs* R320H 0.085 ± 0.013 (p = 0.0005) and P453A 0.060 ± 0.007 (p = 0.062); for Triac: wild-type 0.023 ± 0.001 vs R320H 0.094 ± 0.014 (p < 0.0001) and P453A 0.041 ± 0.004 (p = 0.051)), substantial impairments were seen for others (e.g., for T3: wild-type 0.033 ± 0.004 *vs* G344E 515 ± 82 (p < 0.0001) and P452L 3.8 ± 0.2 (p < 0.0001); for Triac: wild-type 0.023 ± 0.001 vs G344E 391 ± 47 (p < 0.0001) and P452L 6.2 ± 0.3 (p < 0.0001)), while the G432del mutant was not responsive to any concentration of T3 or Triac used. T3 and Triac exhibited similar transcriptional responses across the entire spectrum of mutants, with R316H being a notable exception showing a much stronger effect with Triac than T3 (Fig. 2). Smaller but significant differences were seen for 4 other mutants with S314F functioning slightly better and M310L, R338W and P452L slightly worse on Triac (Supplementary Table S5). We next aimed to confirm the receptor activities of wild-type and mutants in studies with the MAL-TRE. Fig. 3 and Supplementary Table S6 also show variably impaired transcriptional activities of TRβ2 mutants using the MAL-TRE reporter with both T3 and Triac. Using the MAL-TRE reporter gene, our findings were very similar to data obtained with the negatively regulated TSHα-TRE construct, with strongly decreased or absent transcriptional responses for some mutants (e.g., G344E and G432del) and strong (R316H) or weaker (S314F) or worse (R320H) preferential activation of some mutants by Triac (Supplementary Table S6). For most mutants, maximal activity values were similar to wild-type, except for S314F, which showed a significantly lower maximal activity than wild-type, but this was not further explored. Across all mutants, a strong correlation between the IC50 (TSHα-TRE reporter) and EC50 (MAL-TRE reporter) was observed (Supplementary Fig. S2). Our cellular transfection model was not well suited to address ligand-independent receptor activity (Supplementary Fig. S3) or the effects of Triac (and T3) on dominant negative activity (Supplementary Fig. S4, Supplementary Tables S7 and S8), in line with earlier studies.^27^^,^^28^ We performed linear regression analysis between IC50 (TSHα) or EC50 (MAL) values with Triac, and percentage changes in thyroid function (TSH, FT4, Jostel’s index) per dose of Triac in patients carrying the mutations, and found no significant correlations, also not when patients with antithyroid medication were excluded and/or patients carrying receptor mutants that are not expected to respond to Triac *in vivo*.

**Fig. 2:**
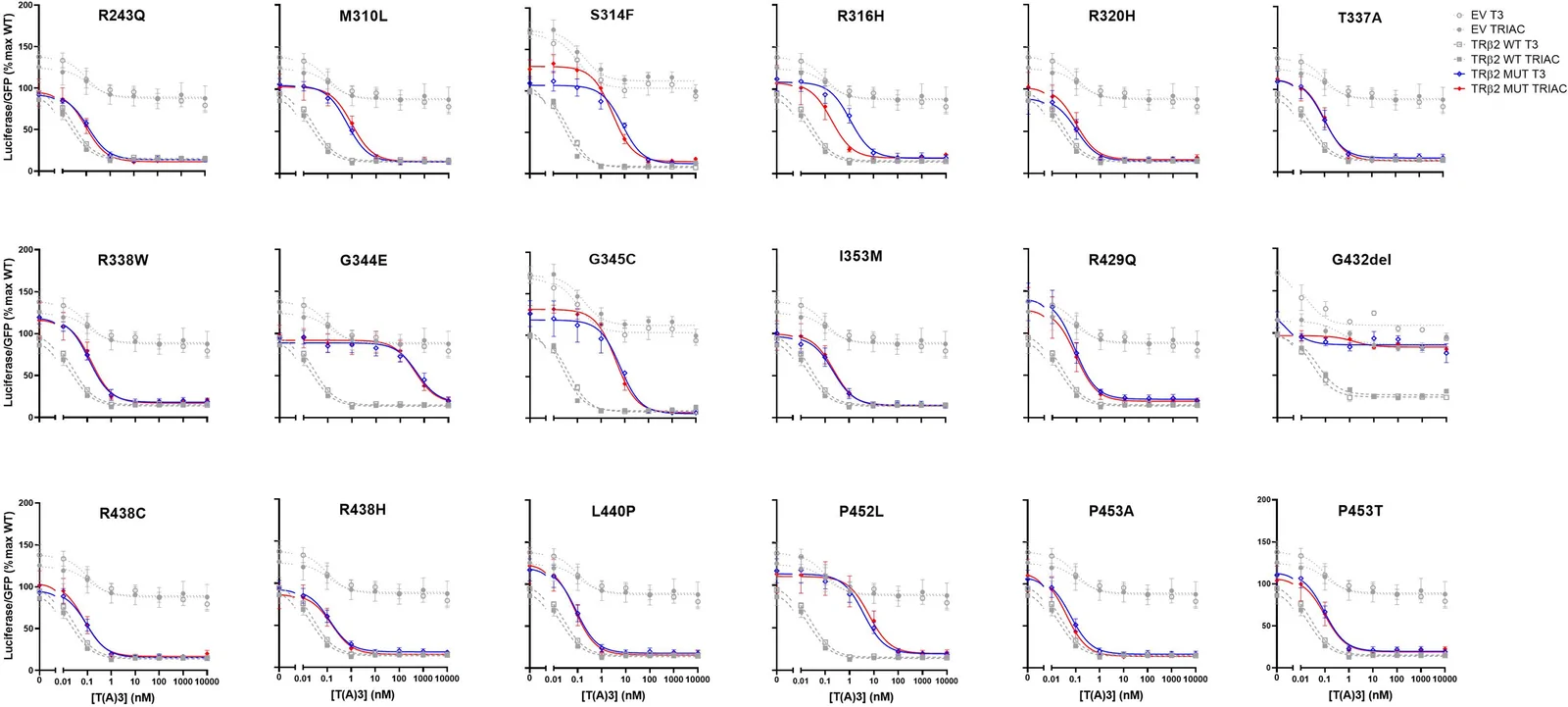
Transcriptional activity of wild-type (WT) and mutant (MUT) TRβ2 (n = 18) on a negative TRE. JEG-3 cells transfected with WT or MUT TRβ2, or empty vector (EV) as control, together with a thyroid hormone responsive luciferase reporter (TSHα-TRE) and exposed to increasing concentrations of T3 and Triac (0–10,000 nM). Each graph shows one MUT TRβ2 in blue (T3) or red (Triac) diamonds, in comparison with EV (circles) and WT TRβ2 (squares) in grey. Open symbols represent T3, closed symbols Triac. Mean ± SEM of at least three experiments performed in triplicate.

**Fig. 3:**
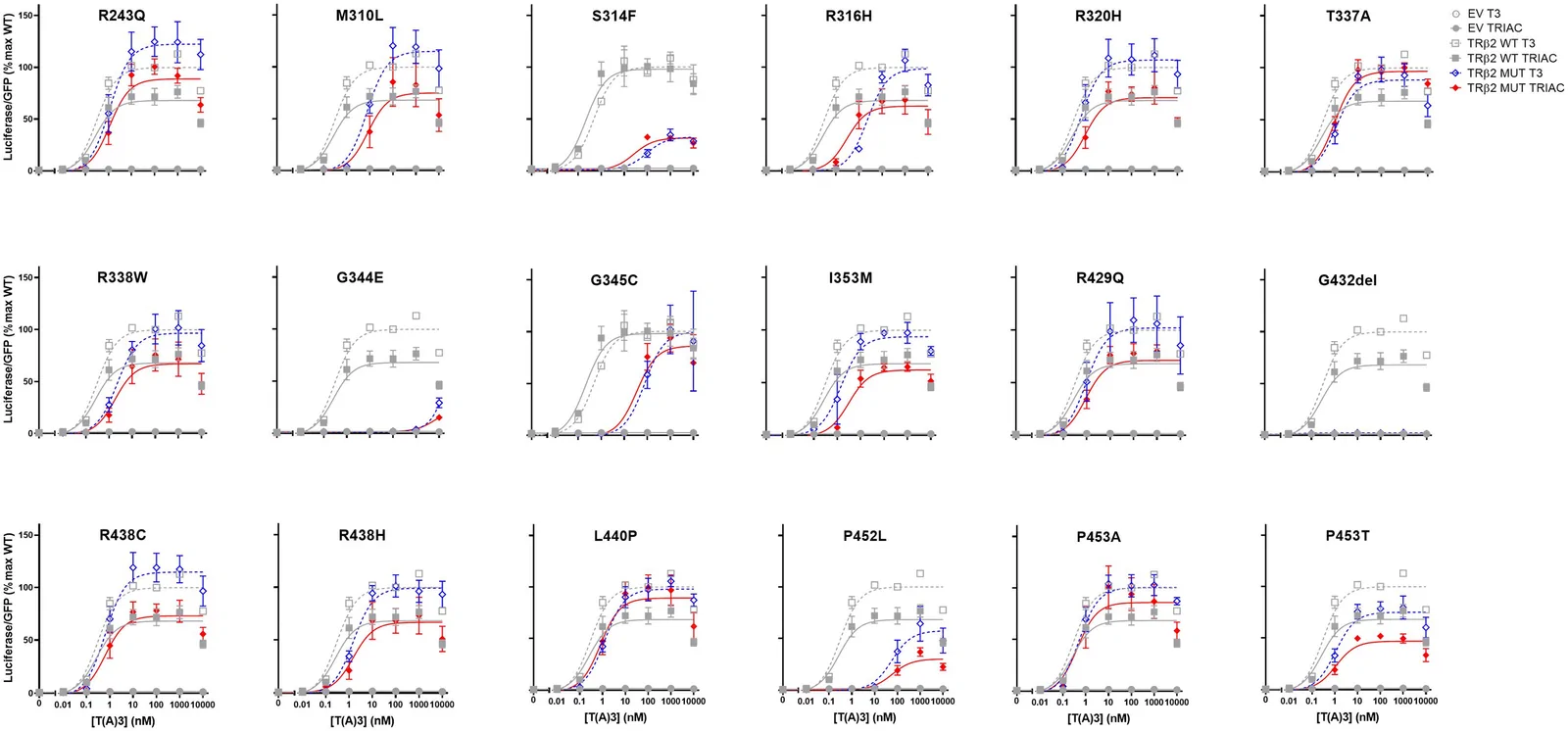
Transcriptional activity of wild-type (WT) and mutant (MUT) TRβ2 (n = 18) on a positive TRE. JEG-3 cells transfected with WT or MUT TRβ2, or empty vector (EV) as control, together with a thyroid hormone responsive luciferase reporter (DR+4-TRE) and exposed to increasing concentrations of T3 and Triac (0–10,000 nM). Each graph shows one MUT TRβ2 in blue (T3) or red (Triac) diamonds, in comparison with EV (circles) and WT TRβ2 (squares) in grey. Open symbols represent T3, closed symbols Triac (TA3). Mean ± SEM of at least three experiments performed in triplicate.

To investigate the effects of Triac on representative mutants *in vivo*, we used an established zebrafish model of RTHβ, injecting wild-type and mutant (R316H, G344E, G432del) TRβ1 or TRβ2 transcripts into zebrafish embryos. To assess the relative expression levels of injected human TRβ transcripts, quantitative real-time PCR analyses were performed at 24, 55, and 120 hpf (Supplementary Fig. S5). Zebrafish express three TRβ-related isoforms with distinct developmental regulation (thrb1S, thrb1L, and thrb2). At 24 hpf, endogenous thrb1S and thrb2 levels were low, whereas thrb1L represented the predominant isoform. At this stage, injected human TRβ transcripts exceeded endogenous thrb1S and thrb2 levels (>2-times) while approximating ∼1- time relative to thrb1L. At 55 hpf, corresponding to HPT-axis differentiation and onset of thyroid hormone synthesis, human TRβ transcript levels declined to approximately 1-time relative to endogenous thrb1S and thrb2, and ∼0.5-time relative to thrb1L. By 120 hpf, exogenous TRβ transcripts were markedly reduced (∼20–30% of endogenous thrb2 and ∼10% of thrb1S/L), confirming the transient nature of receptor overexpression (Supplementary Fig. S5).

At 120 hpf, no differences in *tshba* expression or the volume of T4-producing thyroid follicles were observed between controls and larvae injected with wild-type TRβ1 or TRβ2 (Fig. 4A and F). The injection of all TRβ1 or TRβ2 mutants resulted in variable increases in both tshba-cell volume and T4 synthesis compared to uninjected controls and their wild-type receptor counterparts (Fig. 4C–E, G–K). Of note, in the TRβ2 context, the G344E and G432del mutants produced a stronger activation of the pituitary–thyroid axis than their TRβ1 counterparts. Compared to wild type receptors, injection of TRβ1 and TRβ2 mutants, significantly altered whole-body thyroid hormone concentrations in zebrafish at 120 hpf (Fig. 4L–O). Whole-body TT4, TT3, FT4, and FT3 concentrations were markedly increased in all larvae injected with TRβ1 and TRβ2 mutants, with the strongest effects observed in the TRβ2 context (Fig. 4L–O).

**Fig. 4:**
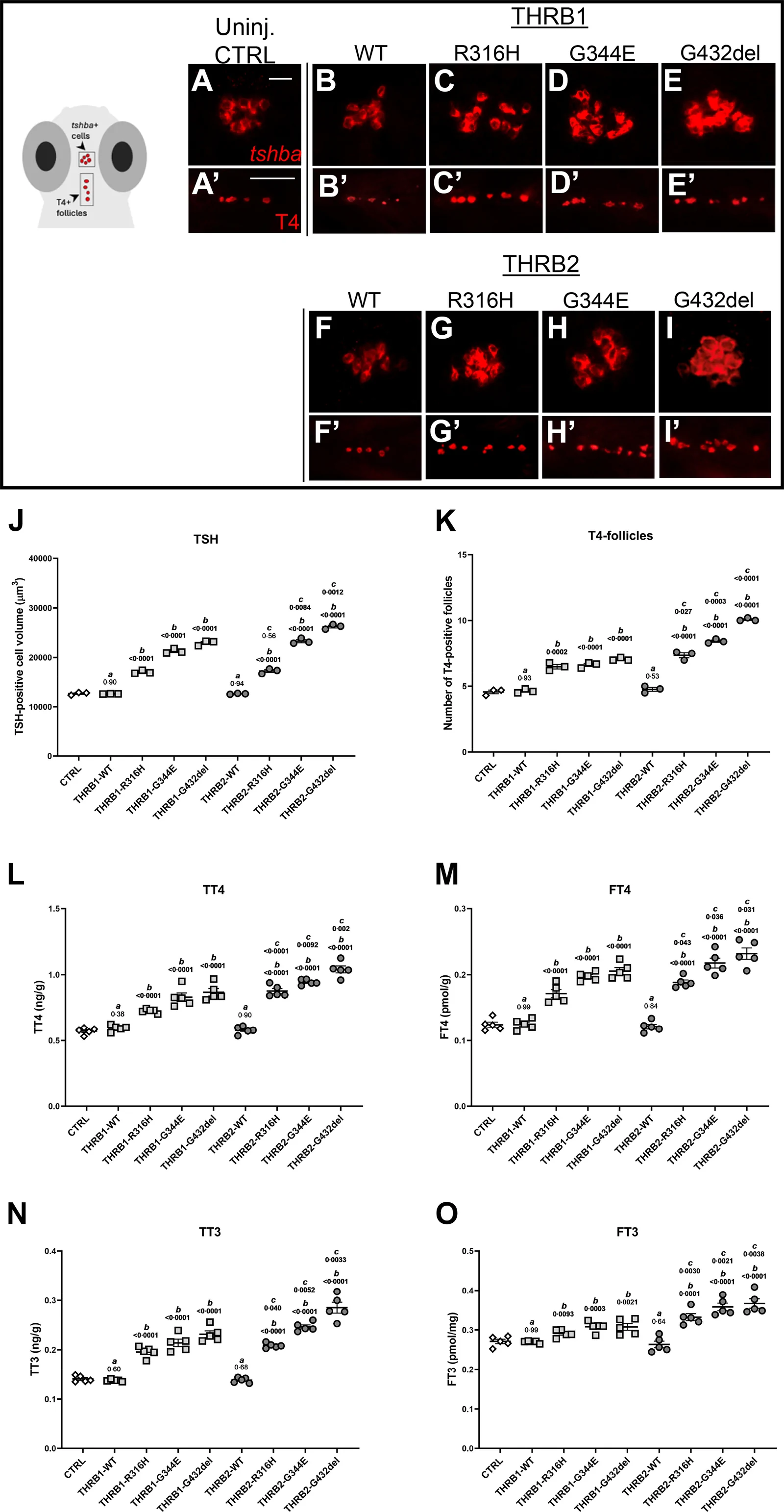
TSH and thyroid hormone profiles in zebrafish larvae injected with wild-type and mutant THRB mRNA constructs. Representative images of FISH of tshba (A–I) and IF of T4 (A’–I’) in CTRL or larvae injected with 50 pg of THRB1 (WT, R316H, G344E, and G432del) and THRB2 (WT, R316H, G344E, and G432del) mRNAs. Fifteen larvae per group were acquired by confocal microscopy, in ventral view, head to the left. Scale bars in CTRL (A and A’): 100 μm. J, quantification of total volume of tshba + -follicles (μm^3^), using confocal microscope analysis on the same area (ROI) and number of sections for each larva. K, Number of T4-positive thyroid follicles. Results are expressed as mean ± SEM of 15 larvae each, derived from three independent injections. (L and M) Whole-body T4 (TT4, ng/g) and free T4 (FT4, pmol/mg) levels, and (N and O) whole-body T3 (TT3, ng/g) and free T3 (FT3, pmol/mg) level. The results are expressed as mean ± SEM of five batch of larvae (approximately 200 each). The comparison between groups was performed One-way ANOVA (a) controls versus WT mRNAs; (b) controls vs WT vs MUT mRNAs; or Unpaired t-test (c) THRB1 MUT vs THRB2 MUT. A p value < 0.05 was considered significant (values in bold).

Next, we evaluated TSH responses in zebrafish expressing wild-type or mutant TRβ2 receptors. The TSH suppression test was performed by treating embryos from 6 to 120 hpf with increasing concentrations (0–20 nM) of T3 or Triac. All mutant-expressing embryos exhibited decreased sensitivity to T3, with the G432del mutant being unresponsive to T3 (Fig. 5A). However, Triac was able to reduce TSH in all mutants (Fig. 5B). Similar effects were seen in zebrafish expressing wild-type or mutant TRβ1 receptors (Supplementary Fig. S6).

**Fig. 5:**
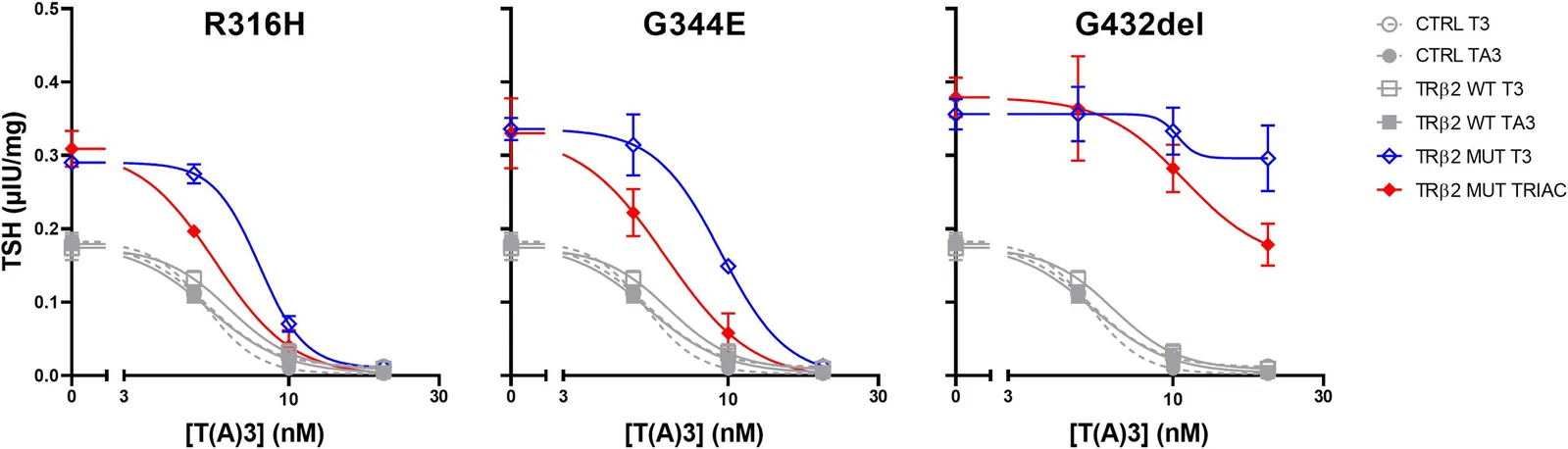
TSH suppression by T3 and Triac in zebrafish larvae injected with wild-type and THRB2 mRNA constructs. Whole-body TSHβ concentration (mlU/mg) in basal condition (1.2% DMSO) or after the administration of increasing doses (5, 10, 20 nM) of T3 or Triac (TA3). Results are expressed as mean ± SEM of three batches of larvae (approximately 200 each).

Finally, we examined the impact of the G344E and R316H mutants, which exhibit differential sensitivity to T3 versus Triac, on the conformation of the ligand binding domain and direct substrate interactions in the T3-liganded (PDB# 3GWS) and Triac-liganded (PDB# 3JZC) TRβ1 crystal structures^23^^,^^24^(Fig. 6A). The G344E variant causes a pronounced change in the structural organization of particularly those domains embedding the outer ring of the T3 molecule (Fig. 6B), including helix 11 and 12 and their connecting loop, which may directly impact substrate binding and co-activator recruitment. In case of T3-liganded TRb1, a pronounced shift in well-known ligand-interacting residues was noticed, including His435, Leu346 and Phe272 (Fig. 6B).^14^ Similar changes were observed in Triac-liganded TRβ1 (Fig. 6C). Together, these changes suggest that the G344E may result in extensive disorganization of the ligand binding domain and molecular surface of the TRβ1 receptor. The R316H variant causes relatively small changes in secondary protein structure, including slight displacement of helix 12, as well as reorientation of several side-chains of ligand-facing residues (Fig. 6D). Consequently, the configuration of the T3 molecule is altered compared to wild-type (RMSD 1.062 A), with compression of the alanine side chain and a slight outward displacement of the T3 outer ring, suggesting that the R316H variant may decrease the size of the ligand-binding pocket. Yet, the distances between T3 and most of its prime interacting residues remain similar, which may explain its relatively preserved ligand binding affinity. These structural changes were less pronounced in Triac-liganded TR, which showed only minimal changes in secondary protein structure and orientation of ligand-facing residues (Fig. 6E). Most pronounced reorientations involve the side chains of Leu330 and Arg320, facing the inner ring and acetic acid moiety of Triac, respectively. Again, the relative distances between Triac and its prime interacting residues remain similar. This also holds for Leu330 and Arg280 as a consequence of slight reorientation of the Triac molecule in the ligand-binding pocket (RMSD 0.834 A compared to wild-type). Taken together, the R316H variant appears to reduce the size of the ligand-binding pocket without major alterations in secondary protein structure and, therefore, it may perfectly accommodate smaller substrate molecules such as Triac.

**Fig. 6:**
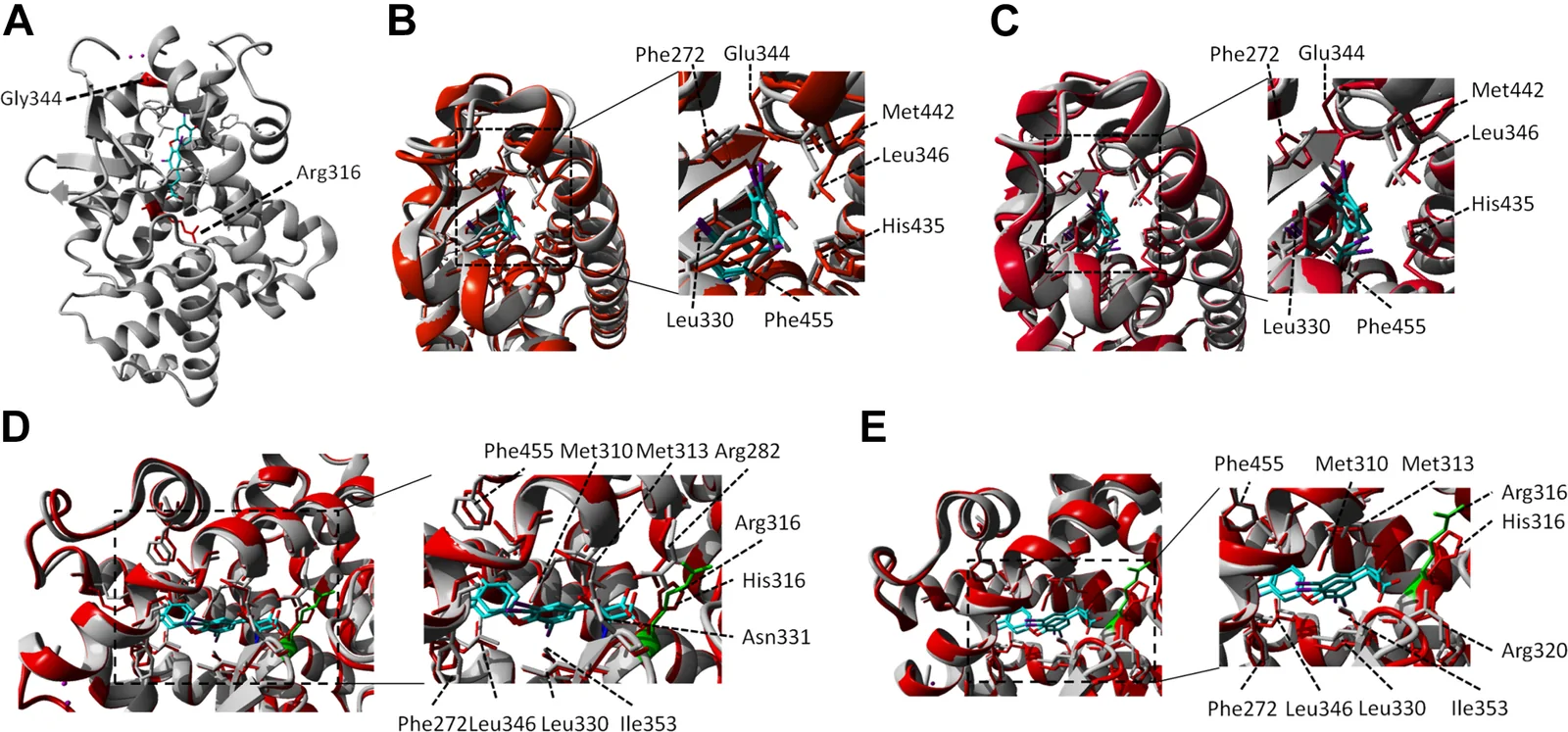
Structural modelling of G344E and R316H variants. (A) Crystal structure of T3-liganded WT TRβ1 (PDB-ID: 3GWS) in which the side-chains of the affected Gly344 and Arg316 are depicted in red. Structural models of the G344E variant (red) in the T3-liganded (B; PDB-ID: 3GWS) and Triac-liganded (C; PDB-ID 3JZC) TRβ1 receptor are presented as an overlay with the respective wild-type crystal structures (grey). Similarly, structural models of the R316H variant (red) in the T3-liganded (D) and Triac-liganded (E) TRβ1 receptor are presented. Depicted are the secondary protein structures and the side chains of all residues with <2.5 Å distance from the ligand molecule. Right subpanels of B-E represent close-ups of the areas within the dashed boxes in the left subpanels.

## Discussion

In this international, multicentre cohort study, we have documented the effectiveness of Triac in the largest intervention cohort of patients with RTHβ together with detailed *in vitro* molecular studies and *in vivo* zebrafish studies, elucidating its underlying mechanisms of action.

A principal finding is that Triac can effectively reduce TSH and FT4 concentrations across patients with RTHβ harbouring a wide range of mutations, in agreement with individual case studies of 16 genetically confirmed cases.^11^ The substantial changes in pituitary resistance and output indices and less outspoken changes in thyroidal output upon Triac treatment indicate the pituitary as the pivotal target for Triac’s actions. The reduction in FT4 concentrations was accompanied by a reduction in heart rate, without negatively impacting on blood pressure, hepatic resistance or bone turnover markers, in line with earlier findings.^12^ RTHβ and MCT8 deficiency are conditions with a substantially elevated mortality rate, likely because of the lifelong elevated thyroid hormone concentrations that negatively impact on different tissues, particularly the cardiovascular system.^7^^,^^29^ As early reduction in thyroid hormone concentrations in Graves’ disease, a condition in which thyroid hormone concentrations are temporarily elevated, is associated with an improved survival,^30^ it suggests that alleviation of the lifelong thyrotoxicosis in RTHβ, and also MCT8 deficiency, could improve cardiovascular morbidity and mortality. Our findings showing a substantial decrease in FT4 concentrations in all patients accompanied by a reduction in heart rate, indicate that Triac might have the potential to reduce clinical negative sequelae in RTHβ, a hypothesis that should be tested in intervention trials.

Integrating the clinical observations with our extensive *in vitro* studies of all mutations present in our cohort, as well as representative mutations in an established zebrafish model provides information about the mechanisms of action of Triac in RTHβ. First, a key finding was the positive effect of Triac on TSH and (F)T4 concentrations in humans and zebrafish with the G344E and G432del mutations, whilst Triac had negligible (for the G432del mutation) or strongly reduced (for the G344E mutation) transcriptional activity with these mutants *in vitro*. These observations can be reconciled if Triac exerts its effects through the wild-type rather than the mutant receptor. Second, at the other end of the mutation spectrum, we noticed positive effects of Triac in humans and zebrafish with the R316H mutation. Our *in vitro* experiments showed a strongly increased transcriptional response of Triac above T3 in cells expressing the R316H mutant. These observations were supported by *in silico* modelling studies showing alterations in the size of the ligand binding pocket that favour binding of the smaller Triac molecule over T3. Assuming that wild-type TRα has a minimal functional role within the hypothalamic-pituitary-thyroid axis, the observed effects of Triac in our homozygous (R316H/R316H) patient can only be mediated via mutant TRβ. Third, the majority of patients harboured mutations that exhibited equally reduced transcriptional responses to both T3 and Triac. We also found that wild-type TRβ was activated similarly by T3 or Triac. Previously, it was reported that some (e.g., R316H, R338W, P453S), but not all (e.g., P453H, G345R, T337del) mutants exhibited slightly increased transcriptional responses to Triac versus T3, similar to the greater affinity of TRβ for Triac over T3.^27^^,^^28^^,^^31^ These discrepancies may reflect differences in experimental design (e.g., use of albumin *vs* stripped serum). Contrary to current belief, Triac may act via different mechanisms in different RTHβ cases, ranging from activating either wild-type receptor or preferentially activating mutant TRb, and possibly a combination of both mechanisms in most mutations. Serum Triac concentrations in treated patients may reach up to 10-times the average concentrations of T3 (unpublished data), having the potential to activate some mutants with IC50 and EC50 values in this concentration range. Moreover, our findings in zebrafish suggest that Triac may accumulate better than T3 in the pituitary. Thus, pharmacokinetics could explain why Triac might be more effective than T3 in such patients. TRβ mutants exhibiting IC50 and EC50 values in the high nM range are unlikely to be activated by Triac. Together with the absence of a clear relationship between mutant and Triac dose and effectiveness, such observations support the notion that activation of the wild-type receptor by this analogue may be a relevant mechanism in many RTHβ cases. Accordingly, we suggest that the effects of Triac in patients with RTHβ might be explained by different mechanisms depending on the mutation-specific properties, with the activation of the wild-type receptor playing a key role in many patients.

Our study also enabled comparison of thyroid function tests of patients carrying a heterozygous versus homozygous R316H mutation. In line with other homozygous cases, the TTSI, a marker of resistance within the HPT axis, was strongly increased in the homozygous R316H/R316H patient compared to the heterozygous wild-type/R316H patient.^26^^,^^32^ This suggests that for many mutants, their dominant negative inhibitory effect is incomplete, enabling residual wild-type receptor action to determine the HPT axis. This observation provides further support for our hypothesis that Triac can act via activation of the wild-type receptor.^33^^,^^34^ This study has several limitations. First, selection bias is likely as symptomatic patients with RTHβ are more likely to receive Triac than those without symptoms. Moreover, patients with RTHβ who stopped Triac therapy because it did not lower thyroid hormone concentrations were not included. Although none of the centres could recall such patients, except for poor adherence, given the retrospective design of the study, such selection bias cannot be excluded. Also, patients exhibiting complaints not well controlled by other means Second, our cohort was genetically and clinically heterogeneous, encompassing cases with concomitant autoimmune thyroid disease and variable use of antithyroid drugs. Although we studied subjects with diverse TRβ mutations, localizing to all three mutational hotspots in the ligand binding domain, not all mutations reported in the literature were tested, formally limiting knowledge of the effectiveness of Triac to the subset of mutations currently reported. Specifically, premature stop mutations seen in severe RTHβ cases were not included, although limited evidence suggests that Triac can be a useful drug treatment.^35^ Third, the retrospective nature of this study precluded us to collect data in a uniform way. Fourth, our cellular overexpression system allowed us to test the mutant receptors for T3 and Triac sensitivity *per se* but was less suited to test the dominant negative activity on WT receptors as present in the heterozygous conditions in patients. Fifth, although activity of the firefly luciferase reporter gene within our constructs can be inhibited (∼2–2.5-fold) by T3,^36^^,^^37^ the greater repression (∼7-fold) of TSHa-LUC reporter gene activity observed with T3 or Triac, indicate that this a valid paradigm to measure hormone-dependent negative transcriptional regulation. Sixth, our findings in untreated zebrafish expressing human mutant TRβ receptors were in line with previous studies, modelling certain aspects of RTHβ.^17^^,^^18^ Although dominant negative effects of human mutant TRβ receptors were clearly present, it not necessarily reflects the exact situation in patients (e.g., endogenous wild-type zebrafish TR forms receptor are present). The increased T4 synthesis in zebrafish expressing mutant TRβ receptors is compatible with the increased T4 production rate and an increased thyroid volume in RTHβ patients. However, the implication of increased tshba-cell volume in the zebrafish expressing mutant TRβ receptors is less clear. Although gland size can change upon (patho)physiological changes,^33^ pituitary size corrected for total intracranial volume was not different in patients with RTHβ versus controls^34^; it is unclear whether TSH-expressing cell volume would be different in patients with RTHβ versus controls.

Different knowledge gaps should be addressed in future studies. The effects of Triac on clinical and biochemical parameters in patients with RTHβ, differentiated for paediatric and adult populations, should be systematically investigated, preferably using interventional clinical trials. Mechanisms underlying negative regulation by T3, dominant negative effects mutant TRβ and any therapeutic intervention to ameliorate the effects of mutant TRβ should be explored, preferably in models with endogenous TR, avoiding overexpression of TRs or other co-factors and/or readouts.

In summary, this collaborative study provides an integrated characterization of the effectiveness and molecular activity of Triac in RTHβ. Our findings indicate that Triac effectively reduces elevated thyroid hormone concentrations in a large cohort of patients with RTHβ harbouring a wide range of different TRβ mutations, providing further support to recommendations for Triac treatment in guidelines by the European Thyroid Association.^38^ From our combination of *in vitro* and *in vivo* observations it can be inferred that Triac can act via different mechanisms depending on the mutation-specific properties, with activation of the wild-type receptor by Triac being an important new insight, relevant to the majority of patients with RTHβ described hitherto who exhibit the heterozygous form of the disorder in which cells express both mutant and wild-type TRβ. Accordingly, our study may inform both clinical management of RTHβ and shape future interventional trials with Triac on clinical and biochemical outcomes in this disorder.

## Contributors

W.L., M.M. and W.E.V. conceived and designed the study and drafted the manuscript. W.L., F.M., S.G., L.O., S.L. and M.M performed experiments. C.M., I.C., Z.M., E.K., R.P., C.O., G.L., M.A., K.C., L.P., and W.E.V. contributed information on patients. All authors (W.L., F.M., C.M., I.C., S.G., Z.M., E.K., R.P., C.O., L.O., S.L., G.L., R.P.P., K.C., L.P., M.M. and W.E.V.) had a critical review of the manuscript. W.E.V. and M.M. accessed and verified underlying data. All authors read and approved the final version of the manuscript.

## Data sharing statement

Because of the rarity of RTHβ, individual participant data beyond that reported here will not be shared, to safeguard patient privacy following (local) data privacy law like the General Data Protection Regulation. Other data are available upon reasonable request from W.E. Visser (w.e.visser@erasmusmc.nl), with the period for response to the access request of one calendar month. The raw data of *in vitro* and *in vivo* studies are available upon request at the following link: https://doi.org/10.34894/OOLMV5.

## Declaration of interests

The Erasmus Medical Centre (Rotterdam, Netherlands), which employs WL, SG, SL, FVG, RP, MM and WEV, receives royalties Egetis Therapeutics (the manufacturer of Triac). None of the authors will benefit personally from any royalties. Egetis Therapeutics had no influence on the conduct or analysis of this study. LP received fees from Egetis Therapeutics for advisory board meetings. CM provides consultancy advice to Egetis Pharmaceuticals and Amgen, received lecture fees from Indonesian Society or Endocrinology Congress, publication fees by IBSA for another publication and travel stipend from American Thyroid Association, is member of monitoring committee LIONNS D2 Study, is the chair of the study review committee on the ReTRIACt study (ClinicalTrials.gov ID NCT05579327), expert endocrine advisor to Health products regulatory authority (HPRA) of Ireland (both are honorary positions) and member of the Executive Committee of the European Thyroid Association (ETA), member of the ETA guideline for Thyroid Hormone Signalling Disorders (until 2024) and lead of the ETA Taskforce on Guidelines for Interference in Immunoassays of Hormones used in Thyroid Function Tests (until Sept 2026), and member of the Executive Committee of the British Thyroid Association The remaining authors declare no competing interests.

## Appendix A Supplementary data

Supplementary Figs. S1–S6 Supplemental Fig. S1. SPINA-GT index in RTHβ patients prior to and during Triac therapy. Supplemental Fig. S2. Correlations between logIC50 and logEC50 values of wild-type and mutant TRβ2 with either T3 (A) or Triac (B). Supplemental Fig. S3. Effect of increasing amounts of unliganded wild-type (WT) and mutant TRβ2 on TSHα promoter activity. JEG3 cells were transfected and assayed as described in the method section. Amounts of receptor plasmid varied from 0 to 20 ng, with total amount of plasmid kept constant at 20 ng by adding additional empty. Mean ± SEM of three experiments performed in triplicate. Statistical significance was tested using a 2-way ANOVA with a Bonferroni post-hoc test. P values for statistically significant differences with WT are indicated. Supplemental Fig. S4. Transcriptional activity of wild-type (WT) and mutant (MUT) TRβ2 (n = 3) on a negative TRE (A) and positive TRE (B). JEG-3 cells transfected with WT or MUT TRβ2, or WT TRβ2 at a 1:1 Ratio, and MUT or empty vector (EV) as control, together with a thyroid hormone responsive luciferase reporter (TSHα-TRE or DR+4-TRE) and exposed to increasing concentrations of T3 and Triac (0 – 10000 nM). Each graph shows one MUT TRβ2 in blue (T3) or red (Triac) diamonds alone or in combination with WT TRβ2 in green (T3) or yellow (Triac) , in comparison with EV (circles) and WT TRβ2 (squares) in grey. Open symbols represent T3, closed symbols Triac. Mean ± SEM of at least three experiments performed in triplicate. Supplemental Fig. S5. Expression levels of zebrafish and human thyroid hormone receptors (TRs). Quantification of endogenous zebrafish and human TRβ isoforms (thrb1S, thrb1L, thrb2) following injection of 50 pg/embryo of WT or mutant THRB1 (A-D) or THRB2 (E-H) transcripts, measured at 24, 55, and 120 hpf. Total RNA was extracted from pools of 15 embryos/larvae per sample. Gene expression levels were calculated as fold change relative to the housekeeping gene eef1a. Data are presented as mean ± SD of biological triplicates. Supplemental Fig. S6. TSH suppression by T3 and Triac in zebrafish larvae injected with wild-type and THRB1 mRNA constructs. Whole-body TSHβ concentration (mlU/mg) in basal condition (1·2% DMSO) or after the administration of increasing doses (5,10,20 nM) of T3 or Triac (TA3). Results are expressed as mean ± SEM of three batches of larvae (approximately 200 each).
