# Supplementary material for: Molecular mechanisms underlying the effectiveness of triiodothyroacetic acid (Triac) in patients with resistance to thyroid hormone β (RTHβ): a multicentre, international, retrospective cohort study
Source: eBioMedicine. 2026 Oct 14;133:106512. doi: 10.1016/j.ebiom.2026.106512 (PMC13683759; doi:10.1016/j.ebiom.2026.106512)
Supplement: Supplementary file 1 [file mmc2.docx]

**Supplementary table 1: FLAG-TRβ2 cloning primers**

|  | Primer sequence (5’→3’) |
| --- | --- |
| Forward: | GTAGAATTCTGGCCGAAATGGACTACAAAGACGATGACGACAAGATGAACTACTGTATGCAAGAAATATATGAAG |
| Reverse: | CTATCTAGACTAATCCTCGAACACTTCCAAGAAC |

**Supplementary table 2: Mutagenesis primers**

| Mutation | Base change |  | Primer sequence (5’→3’) |
| --- | --- | --- | --- |
| R243Q | c.728G>A | **Forward:**  **Reverse:** | CCACTGGAAGCAAAAACAGAAATTCCT  AGGAATTTCTGTTTTTGCTTCCAGTGG |
| M310L | c.928A>C | **Forward:**  **Reverse:** | CTCAAAGGCTGCTGCCTGGAGATCATGTCC  GGACATGATCTCCAGGCAGCAGCCTTTGAG |
| S314F | c.941C>T | **Forward:**  **Reverse:** | GCTGCTGCATGGAGATCATGTTCCTTCGCGCTG  CAGCGCGAAGGAACATGATCTCCATGCAGCAGC |
| R316H | c.947G>A | **Forward:**  **Reverse:** | GATCATGTCCCTTCACGCTGCTGTGCGCT  AGCGCACAGCAGCGTGAAGGGACATGATC |
| R320H | c.959G>A | **Forward:**  **Reverse:** | CTTTCTGGGTCATAGTGCACAGCAGCGCGAA  TTCGCGCTGCTGTGCACTATGACCCAGAAAG |
| T337A | c.1009A>G | **Forward:**  **Reverse:** | GGAAATGGCAGTGGCACGGGGCCAGCT  AGCTGGCCCCGTGCCACTGCCATTTCC |
| R338W | c.1012C>T | **Forward:**  **Reverse:** | GGGAAATGGCAGTGACATGGGGCCAGC  GCTGGCCCCATGTCACTGCCATTTCCC |
| G344E | c.1031G>A | **Forward:**  **Reverse:** | GGGGCCAGCTGAAAAATGAGGGTCTTGGGGTG  CACCCCAAGACCCTCATTTTTCAGCTGGCCCC |
| G345C | c.1033G>T | **Forward:**  **Reverse:** | CCAGCTGAAAAATGGGTGTCTTGGGGTGGTGTC  GACACCACCCCAAGACACCCATTTTTCAGCTGG |
| I353M | c.1059C>G | **Forward:**  **Reverse:** | GGTGTCAGACGCCATGTTTGACCTGGGCATG  CATGCCCAGGTCAAACATGGCGTCTGACACC |
| R429Q | c.1286G>A | **Forward:**  **Reverse:** | AAGGTGACAGATCTGCAGATGATAGGA  AAGGTGACAGATCTGCAGATGATAGGA |
| G432del | c.1294-1296*del*GGA | **Forward:**  **Reverse:** | TCTGCGGATGATAGCCTGCCATGCCAGC  GCTGGCATGGCAGGCTATCATCCGCAGA |
| R438C | C1312C>T | **Forward:**  **Reverse:** | CCTGCCATGCCAGCTGCTTCCTGCACATG  CATGTGCAGGAAGCAGCTGGAATGGCAGG |
| R438H | c.1313G>A | **Forward:**  **Reverse:** | CTGCCATGCCAGCCACTTCCTGCACATGA  TCATGTGCAGGAAGTGGCTGGCATGGCAG |
| L440P | c.1319T>C | **Forward:**  **Reverse:** | GCCAGCCGCTTCCCGCACATGAAGGTG  CACCTTCATGTGCGGGAAGCGGCTGGC |
| P452L | c.1355C>T | **Forward:**  **Reverse:** | CCCCACAGAACTCTTCCTCCCTTTGTTCTTGGAAG  CTTCCAAGAACAAAGGGAGGAAGAGTTCTGTGGGG |
| P453A | c.1357C>G | **Forward:**  **Reverse:** | CCACAGAACTCTTCCCCGCTTTGTTCTTGGAAGTG  CACTTCCAAGAACAAAGCGGGGAAGAGTTCTGTGG |
| P453T | c.1357C>A | **Forward:**  **Reverse:** | CCACAGAACTCTTCCCCACTTTGTTCTTGGAAGTG  CACTTCCAAGAACAAAGTGGGGAAGAGTTCTGTGG |

**Supplementary table 3: Zebrafish qPCR primers**

| Transcript |  | Primer sequence (5’→3’) |
| --- | --- | --- |
| THRB1 | **Forward:**  **Reverse:** | ATGACTCCCAACAGTATGAC ACATTCCTACTAGCTTCCAG |
| THRB2 | **Forward:**  **Reverse:** | CCAGCTGCCGGCAGCAATTG TACTGGGCACAGCCTGAGCA |
| thrb1S | **Forward:**  **Reverse:** | AATTCCTGCCAGAAGACATT AATCCACGACACGAGTGAT |
| thrb1L | **Forward:**  **Reverse:** | AATTCCTGAGTGCAGTGGG TCCACGACACGAGTGATGG |
| thrb2 | **Forward:**  **Reverse:** | ATGAATTACTGCATGTCAGACA TGTAACGGCTGTGGTTCA |

| **Experimental** | **Developmental** | **Experimental** | **Experimental** | **Biological** | **Inclusion / exclusion criteria** | **Randomization** | **Sample size rationale** |  |
| --- | --- | --- | --- | --- | --- | --- | --- | --- |
| **setting** | **stage** | **groups** | **unit** | **replicates** |  |  |  |  |
| qRT-PCR expression profiling (human vs endogenous TRβ) | 24, 55, 120 hpf | WT TRβ1/2; MUT TRβ1/2 | Pool of 15 embryos/larvae | 3 independent injections | Homogenous tracer distribution, normal morphology / severe delay, necrosis or malformation | Embryos from multiple clutches randomly allocated; pools assembled by random selection | Pool size defined by RNA yield and stable Ct variability |  |
| FISH (tshba) | 120 hpf | Uninjected; WT TRβ1/2; MUT TRβ1/2 | Individual larvae (n=15) | 3 independent injections | Same as above | Random selection for imaging | Based on imaging reproducibility and prior optimization. Predefined acquisition settings. |  |
| IF (T4 follicles) | 120 hpf | Uninjected; WT TRβ1/2; MUT TRβ1/2 | Individual larvae (n=15) | 3 independent injections | Same as above | Random selection for imaging | Based on imaging reproducibility and prior optimization. Predefined acquisition settings. |  |
| ELISA (Hormone quantification) | 120 hpf | Uninjected; WT TRβ1/2; MUT TRβ1/2 | Pool of ~200 larvae | 5 pools obtained from > 20 independent injections | Same as above | Pool assembled by random selection | Pool size required to meet ELISA detection limits. Predefined plate-reader quantification settings. |  |
| TSH suppression assay (T3/Triac dose-response) | 120 hpf | Uninjected; WT TRβ1/2; MUT TRβ1/2 | Pool of ~200 larvae | 3 pools obtained from > 5 independent injections | Same as above | Pool assembled by random selection | Pool size required to meet ELISA detection limits. Predefined plate-reader quantification settings. |  |
|  |  |  |  |  |  |  |  |  |

**Supplemental table 4: Experimental design and key characteristics of zebrafish studies**

**Supplemental table 5: Transcriptional activity of WT and MUT TRβ2 with T3 or Triac on TSHα-TRE.**

|  | TRβ2 | LogIC50 Triac*^#^*  IC50 (nM) | LogIC50 T3*^#^*  IC50 (nM) | LogIC50 (Triac-T3)*^†^*  Fold IC50(Triac/T3) | P*^‡^*  value |
| --- | --- | --- | --- | --- | --- |
|  | WT | -1·62±0·02  0·023±0·001 | -1·48±0·07  0·033±0·004 | -0·14±0·05  0·72 | - |
|  | R243Q | -1·02±0·06  0·10±0·01 | -0·92±0·03  0·12±0·00 | -0·10±0·08  0·80 | 0·99 |
|  | M310L | -0·00±0·09  1·0±0·1 | -0·21±0·07  0·61±0·06 | 0·21±0·04  1·61 | **0·0012** |
|  | S314F | 0·50±0·02  3·1±0·1 | 0·91±0·02  8·1±0·3 | -0·41±0·03  0·39 | **0·027** |
|  | R316H | -0·73±0·02  0·19±0·01 | 0·04±0·06  1·1±0·1 | -0·77±0·04  0·17 | **<0·0001** |
|  | R320H | -1·04±0·10  0·091±0·014 | -1·08±0·08  0·085±0·010 | 0·04±0·09  1·12 | 0·25 |
|  | T337A | -1·00±0·08  0·10±0·01 | -1·06±0·08  0·087±0·010 | 0·06±0·02  1·14 | 0·17 |
|  | R338W | -0·82±0·05  0·15±0·01 | -0·92±0·03  0·12±0·01 | 0·10±0·06  1·27 | **0·048** |
|  | G344E | 2·59±0·08  391±47 | 2·71±0·10  515±82 | -0·12±0·09  0·77 | 1·00 |
|  | G345C | 0·64±0·05  4·4±0·3 | 0·85±0·08  7·1±0·9 | -0·21±0·03  0·62 | 1·00 |
|  | I353M | -0·63±0·03  0·23±0·01 | -0·68±0·09  0·21±0·02 | 0·05±0·11  1·14 | 0·20 |
|  | R429Q | -1·03±0·07  0·094±0·010 | -1·06±0·04  0·087±0·006 | 0·04±0·03  1·09 | 0·30 |
|  | G432del | -  - | -  - | -  - | - |
|  | R438C | -1·14±0·03  0·073±0·004 | -1·04±0·04  0·091±0·006 | -0·10±0·02  0·80 | 0·99 |
|  | R438H | -0·83±0·04  0·15±0·01 | -0·89±0·07  0·13±0·01 | 0·06±0·04  1·15 | 0·16 |
|  | L440P | -1·13±0·07  0·075±0·008 | -1·07±0·05  0·085±0·006 | -0·05±0·04  0·89 | 0·97 |
|  | P452L | 0·79±0·04  6·2±0·3 | 0·58±0·03  3·8±0·2 | 0·21±0·01  1·64 | **0·0009** |
| P453A | | -1·39±0·07  0·041±0·004 | -1·22±0·08  0·060±0·007 | -0·17±0·11  0·69 | 1·00 |
|  | P453T | -1·03±0·09  0·094±0·013 | -1·01±0·03  0·10±0·00 | -0·02±0·09  0·96 | 0·77 |

\# LogIC50 values were extracted from non-linear curve fitting of individual experiments, averaged and antilog transformed to IC50 values.

† Log IC50 values for T3 were subtracted from LogIC50 values for Triac and antilog transformed for fold IC50 for Triac over T3.

‡ P values indicate significance of differences in fold IC50 Triac over T3 for mutants compared to WT as one-way ANOVA with Dunnett’s multiple comparisons test.

Values could not be extracted for all Triac curves for G344E or any curve for G432del due to incomplete curves.

Mean ± SEM of at least three experiments performed in triplicate.

**Supplemental table 6: Transcriptional activity of WT and MUT TRβ2 with T3 or Triac on MAL-TRE.**

| TRβ2 | LogEC50 Triac*^#^*  [EC50 (nM)] | LogEC50 T3*^#^*  [EC50 (nM)] | LogEC50 (Triac/T3)*^†^*  [Fold EC50] | P*^‡^*  value |
| --- | --- | --- | --- | --- |
| WT | -0·61±0·06  0·25±0·02 | -0·41±0·07  0·39±0·04 | -0·20±0·09  0·64 | - |
| R243Q | 0·07±0·16  1·2±0·3 | 0·10±0·18  1·3±0·4 | -0·03±0·03  0·93 | 0·79 |
| M310L | 0·95±0·17  9·3±2·4 | 0·86±0·23  7·5±1·5 | 0·09±0·05  1·23 | 0·13 |
| S314F | 1·42±0·05  26±2 | 1·95±0·16  92±22 | -0·53±0·21  0·29 | **0·041** |
| R316H | 0·50±0·13  3·2±0·7 | 1·36±0·09  23±3 | -0·86±0·06  0·14 | **<0·0001** |
| R320H | -0·01±0·19  1·0±0·3 | -0·17±0·12  0·69±0·13 | 0·16±0·07  1·45 | **0·024** |
| T337A | 0·04±0·13  1·1±0·2 | -0·02±0·08  0·97±0·12 | 0·06±0·10  1·15 | 0·22 |
| R338W | 0·29±0·15  2·0±0·5 | 0·33±0·15  2·2±0·5 | -0·04±0·01  0·92 | 0·81 |
| G344E | nd  nd | nd  nd | nd  nd | - |
| G345C | 1·55±0·05  35±3 | 1·97±0·11  95±15 | -0·43±0·09  0·38 | 0·35 |
| I353M | 0·52±0·12  3·3±0·6 | 0·47±0·09  3·0±0·4 | 0·05±0·03  1·12 | 0·27 |
| R429Q | -0·02±0·12  0·96±0·18 | 0·03±0·13  1·1±0·2 | -0·05±0·03  0·88 | 0·91 |
| G432del | nd  - | nd  - | -  - | - |
| R438C | -0·19±0·19  0·67±0·19 | -0·20±0·12  0·64±0·11 | 0·01±0·07  1·02 | 0·50 |
| R438H | 0·20±0·14  1·6±0·3 | 0·17±0·10  1·5±0·2 | 0·03±0·05  1·08 | 0·35 |
| L440P | -0·09±0·16  0·83±0·20 | 0·09±0·07  1·2±0·1 | -0·18±0·10  0·66 | 1·00 |
| P452L | 1·79±0·09  62±9 | 1·78±0·10  61±10 | 0·01±0·02  1·02 | 0·50 |
| P453A | -0·31±0·17  0·51±0·13 | -0·28±0·10  0·53±0·08 | -0·03±0·07  0·94 | 0·75 |
| P453T | 0·06±0·12  1·2±0·2 | 0·03±0·12  2·0±0·2 | 0·02±0·01  1·06 | 0·41 |

\# LogEC50 values were extracted from non-linear curve fitting of individual experiments, averaged and antilog transformed to EC50 values.

† Log EC50 values for T3 were subtracted from LogEC50 values for Triac and antilog transformed for fold EC50 for Triac over T3.

‡ P values indicate signifcance of differences in fold EC50 Triac over T3 for mutants compared to WT as one-way ANOVA with Dunnett’s multiple comparisons test.

Values could not be extracted for G344E and G432del due to incomplete curves.

Mean ± SEM of at least three experiments performed in triplicate.

**Supplemental table 7: Transcriptional activity of WT and MUT TRβ2 only or in combination with WT, with T3 or Triac on TSHα-TRE.**

| TRβ2 | LogIC50 Triac*^#^*  IC50 (nM) | LogIC50 T3*^#^*  IC50 (nM) | LogIC50 (Triac/T3)*^†^*  Fold IC50 | P*^#^*  value |
| --- | --- | --- | --- | --- |
| WT/WT | -1.69±0.10  0.021±0.003 | -0.98±0.35  0.11±0.01 | -0.71±0.18  0.20 | - |
| R316H/R316H | -0.37±0.08  0.43±0.05 | 0.08±0.12  1.2±-0.1 | -0.45±0.12  0.36 | 0.83 |
| R316H/WT | -1.59±0.18  0.027±0.007 | -0.89±0.12  0.13±0.02 | -0.69±0.11  0.22 | 1.00 |
| G344E/G344E | 2.15±0.15  145±33 | 2.54±0.07  352±41 | -0.39±0.16  0.42 | 0.71 |
| G344E/WT | -1.53±0.13  0.030±0.006 | -0.84±0.05  0.15±0.01 | -0.69±0.08  0.20 | 1.00 |
| G432del/G432del | nd  nd | nd  nd | nd  nd | - |
| G432del/WT | -1.57±0.27  0.029±0.013 | -0.76±0.04  0.17±0.01 | -0.82±0.26  0.16 | 0.99 |

\# LogIC50 values were extracted from non-linear curve fitting of individual experiments, averaged and antilog transformed to IC50 values.

† LogIC50 values for T3 were subtracted from LogIC50 values for Triac and antilog transformed for fold IC50 for Triac over T3.

‡ P values indicate signifcance of differences in fold IC50 Triac over T3 for mutants compared to WT as one-way ANOVA with Dunnett’s multiple comparisons test.

Values could not be extracted for G432del/G432del due to incomplete curves.

Mean ± SEM of three experiments performed in triplicate.

**Supplemental table 8: Transcriptional activity of WT and MUT TRβ2 only or in combination with WT, with T3 or Triac on MAL-TRE.**

| TRβ2 | LogEC50 Triac*^#^*  EC50 (nM) | LogEC50 T3*^#^*  EC50 (nM) | LogEC50 (Triac/T3) *^†^*  Fold EC50 | P*^#^*  value |
| --- | --- | --- | --- | --- |
| WT/WT | -0.51±0.36  0.36±0.21 | -0.09±0.35  0.95±0.54 | -0.42±0.10  0.38 | - |
| R316H/R316H | 0.92±0.14  8.4±1.8 | 1.71±0.06  52±5 | -0.80±0.20  0.17 | 0.30 |
| R316H/WT | 0.01±0.30  1.1±0.55 | 0.70±0.25  5.4±2.1 | -0.69±0.11  0.21 | 0.86 |
| G344E/G344E | nd  nd | nd  nd | nd  nd | - |
| G344E/WT | -0.087±0.13  0.83±0.16 | 0.07±0.19  1.2±0.34 | -0.15±0.28  0.35 | 0.99 |
| G432del/G432del | nd  nd | nd  nd | nd  nd | - |
| G432del/WT | 0.25±0.51  2.4±2.2 | -0.02±0.16  0.98±0.25 | 0.27±0.37  1.34 | 0.69 |

\# LogEC50 values were extracted from non-linear curve fitting of individual experiments, averaged and antilog transformed to EC50 values.

† LogEC50 values for T3 were subtracted from LogEC50 values for Triac and antilog transformed for fold EC50 for Triac over T3.

‡ P values indicate signifcance of differences in fold EC50 Triac over T3 for mutants compared to WT as one-way ANOVA with Dunnett’s multiple comparisons test.

Values could not be extracted for G344E/G344E and G432del/G432del due to incomplete curves.

Mean ± SEM of at least four experiments performed in triplicate.
