# Supplementary material for: Molecular mechanisms underlying the effectiveness of triiodothyroacetic acid (Triac) in patients with resistance to thyroid hormone β (RTHβ): a multicentre, international, retrospective cohort study
Source: eBioMedicine. 2026 Oct 14;133:106512. doi: 10.1016/j.ebiom.2026.106512 (PMC13683759; doi:10.1016/j.ebiom.2026.106512)
Supplement: Supplementary file 2 [file mmc3.docx]

Supplemental Data

for

Molecular mechanisms underlying the effectiveness of triiodothyroacetic acid (Triac) in patients with resistance to thyroid hormone β (RTHβ): a multicentre, international, retrospective cohort study

**Reagent Validation File**

xxx
